# 1108. Case Study. Pediatric Burn Care Treatment Using Mini Automated Autologous Cell Harvesting Device: A Tertiary Case Study

**DOI:** 10.1093/jbcr/irag033.212

**Published:** 2026-04-06

**Authors:** Katrina L Weaver

**Affiliations:** Logan Health Children's, Kalispell, MT

## Abstract

**Patient Presentation (age range, injury details, relevant history):**

A 5-year-old male sustained a 4% TBSA mixed-depth flame burn to the lower back and right upper extremity while camping. The closest ABA verified burn center was located multiple states away. Parents requested to stay close to home and wanted the fastest treatment to healing since the start of school was imminent.

**Clinical Challenges:**

Pediatric burn injuries pose unique challenges, particularly in rural regions where verified burn centers may require air transfer. For children, timely healing and strong family support are critical to recovery, promoting early return to school and activities of daily living (ADLs). Adoption of new technologies enables smaller tertiary care centers to keep patients with small TBSA burns close to their families and communities, while optimizing outcomes. The mini automated autologous cell harvesting device (MA-ACHD) generates a skin cell suspension autograft (SCSA) that, when combined with meshed split-thickness skin grafts (mSTSG), supports effective wound healing. Designed for wounds up to 480 cm^2^, the MA-ACHD produces the same quality suspension as the larger automated system while allowing surgeons to allocate resources appropriately and avoid unnecessary waste.

**Management Approach:**

On post-injury day 5, he underwent surgical excision and grafting. A split-thickness skin graft was harvested, meshed 3:1, applied to the full-thickness burn on the lower back, and sprayed with SCSA. Remaining mixed-depth wounds were sprayed with MA-ACHD prepared SCSA. The graft was secured with sutures and dressed with a non-adherent dressing and negative pressure wound therapy.

**Outcomes:**

The patient was discharged on postoperative day (POD) 3. By POD 7, 75% re-epithelialization was observed. By POD 10, complete re-epithelialization was observed, yielding excellent functional results. According to parent report, he resumed normal activities prior to returning to school on POD 14.

Figure 1. Images of the full-thickness flame injury on the patient’s lower back at a) presentation, B) postoperative day (POD) 10, and C) POD 18, showing progression of re-epithelialization with MA-ACHD-prepared SCSA ± meshed split-thickness skin graft.

**Lessons Learned:**

The MA-ACHD was easy to use and produced a reliable suspension, enabling local treatment of a small TBSA burn. The patient achieved complete re-epithelialization by POD 10 and returned to normal activities and school within two weeks. This experience highlights how the device supports efficient resource use and allows patients to remain close to home.

**Applicability to Practice:**

Integrating advanced technologies helps patients remain in their communities, supports continuity of care, and maintains strong outcomes. The MA-ACHD is particularly suited for wounds up to 480 cm^2^, giving surgeons the ability to match device choice to wound size, which is especially critical for pediatric patient populations. This reduces waste, promotes efficient resource allocation, and lowers costs, while sustaining high standards of burn care in resource-limited settings.

